# Effect of variety and drying temperature on physicochemical quality, functional property, and sensory acceptability of dried onion powder

**DOI:** 10.1002/fsn3.707

**Published:** 2018-07-20

**Authors:** Muhaba Seifu, Yetenayet B. Tola, Ali Mohammed, Tessema Astatkie

**Affiliations:** ^1^ Ethiopian Institute of Agricultural Research Debre Zeit Agricultural Research Center Debre Zeit Ethiopia; ^2^ Jimma University College of Agriculture and Veterinary Medicine Jimma Ethiopia; ^3^ Faculty of Agriculture Dalhousie University Truro NS Canada

**Keywords:** drying, postharvest, pungency, storage life, vitamin C

## Abstract

In Ethiopia, onion is one of the most important vegetables/spices produced as a source of cash income and for flavoring foods. However, postharvest loss during storage remains a major challenge. In this study, the effects of variety (Bombay red, Qellafo and Sweet carolin) and drying temperature (Fresh, 50, 60, 70, 80, and 90°C) on physicochemical quality, functional property, and sensory acceptability of dried onion powder were determined. The results indicated that total color change of Bombay red was not affected by temperature, but Qellafo and Sweet carolin varieties showed an increase in color change as drying temperature increases. Bulk density, shrinkage ratio, and water hydration capacity increased with increasing temperature for all three varieties. The degradation of vitamin C, pyruvic acid, and desired sensory attributes increased with increasing oven drying temperature. All in all, Qellafo dried at 70°C for 5 hr was found to be desirable for production of dehydrated onion powder. The findings of this study will allow the identification of the best of the three commonly grown onion varieties in Ethiopia, and the preferred temperature for production of dried onion with minimal negative effect on physicochemical, functional, nutritional, and sensory properties.

## 1 INTRODUCTION

In Ethiopia, onion (*Allium cepa* L.) is the most important vegetable crop used as a spice and food ingredient due to its aroma, flavor, and pungency. It is also a widely used crop globally, primarily due to its several health benefits. It has wound healing, anti‐inflammatory, antioxidant, and antimicrobial properties as well as trypsin and tyrosinase inhibitory properties (Sharma, Gairola, Sharma, & Gaur, 2014). Also, in vivo clinical study on people with hypertrophic scars or keloids showed that topical applications of a gel containing onion extract, pentaglycan, and allantoin are useful in reducing neoangiogenesis in hypertrophic scars and keloids, resulting in clinical improvement of skin lesions (Campanati et al., 2010). An extract of red onion peel was also found to have a strong phosphodiesterase 5A inhibitory activity, which is important for treatment of erectile dysfunction (Lines & Ono, 2006). Besides, onion was one of the medicinal plants recommended to cure erectile dysfunction by medieval Persian practitioners (Ghadiri & Gorji, 2004).

In Ethiopia, smallholder farmers produce onion mainly as a cash crop and for flavoring the local stew. It is believed to be one of the most consumed vegetables in Ethiopian traditional foods than any other vegetables (Lemma & Shemelis, 2003). However, the postharvest loss (PHL) of onion is quite serious in the country due to lack of appropriate handling, storing, and value addition techniques. The bulky nature of fresh onions does also create problems during transportation and storage. At ambient storage environment (20°C, 75%–80% relative humidity), bulbs can be stored for 20 days, but under low temperature storage (0°C, 75%–80% relative humidity), bulbs can be stored for more than 5 months (Thompson, 1996). However, low temperature storage facility is not affordable by small scale farmers in developing countries. According to Daniels and Fors (2015), estimated postharvest loss of onion is 13% due to different factors. Due to fear of spoilage and loss on the field during peak harvesting season, producers are often obliged to sell the produce at uneconomically low price. On the other hand, the limited supply of onions during the off‐peak seasons creates shortage of onion in the market that results in high prices. During market glut, the price of onion at the farm gate could be between 0.04 and 0.074 USD (1 USD = 27 ETB) per kg while during the off season and festivals, it could increase to between 0.59 and 0.74 USD (personal observation), which is almost 10 times the price during the peak harvest. To mitigate this problem, producers commonly use sun drying as a traditional method of reducing onion moisture to increase storage life. But, this method results in an erratic and less efficient drying, which eventually leads to less quality product, and could enable the occurrence of mycotoxins due to the growth of mesospheric mycotoxin‐producing fungi. Thus, under such circumstances, determining a more efficient and cost‐effective drying method that insures better quality finished product with acceptable physicochemical, functional, nutritional, and sensory attributes is important. As stated in Jayathunge and Illeperuma (2001), optimized drying reduces wastage, reduces volume, minimizes transportation and packaging cost and increases the availability of powdered onion throughout the year as an ingredient for food processing industries and home consumption. Therefore, the aim of this work was to study the effect of variety and drying temperature on quality attributes of dried onion powder.

## 2 MATERIALS AND METHODS

### 2.1 Description of the experimental site

The experiment was carried out at the Postharvest management laboratory of Jimma University College of Agriculture and Veterinary Medicine (JUCAVM) in Jimma, Ethiopia.

### 2.2 Sample collection and preparation

Three onion varieties (Bombay red, Qellafo, and Sweet carolin) were procured from local farmers near the town of Zeway, in central rift valley of Ethiopia. Before drying, bulbs were washed; roots were removed, peeled, and sliced to 5‐mm thickness as described in Mitra, Shrivastava, and Rao (2011).

### 2.3 Drying process

The sliced onions were placed inside hot air oven (Model: Leicester, LE67 5FT, UK) at a specific temperature until a constant mass was achieved. As higher temperature needs shorter time, and based on a preliminary study, drying times were set at 2.30, 4, 5, 6, and 7.30 hr for the samples dried at 50, 60, 70, 80, and 90°C, respectively. Then, the dried samples were cooled inside desiccators to avoid condensation of moisture and rehydration. Then, samples cooled to room temperature were ground into powder and packed in high‐density polyethylene bags and stored at ambient atmospheric condition during the study period.

### 2.4 Experimental design

The experiment was carried out as two‐factor factorial design with three replications. The first factor was onion variety with three levels (Bombay red, Qellafo, and Sweet carolin), and the second factor was oven drying temperature with six levels (Fresh (control), 50, 60, 70, 80, and 90°C).

### 2.5 Determination of physical and functional properties

#### 2.5.1 Total color change (ΔE)

The total color change of each sample was measured using color chromameter (Model: Accu‐probe HH06M, America). Color measurements were recorded using Hunter L*, a*, and b* scale (Hassani & Sharifi, 2012) after the required white color calibration. Total color change was calculated to estimate the extent of change in color of dried onion samples using Equation (1).

(1) $$\Delta E=\sqrt{\left(L_{o}-L^{*}\right)+\left(a_{o}-a^{*}\right)+\left(b_{o}-b^{*}\right)}$$

where, ΔE = total color change, *L*_o_, *a*_o_, and *b*_o_ are color parameters of fresh onion varieties, L*, a*, and b* color parameters of dried onion powder.

#### 2.5.2 Bulk density (BD)

Bulk density was determined by the method described in Goula and Adamopoulos (2005) and calculated as:

(2) $$\text{BD}\;\left(g/{\text{cm}}^{3}\right)=\frac{\text{Weight of dried sample}}{\text{Volume of dried sample after tapping}}$$

#### 2.5.3 Shrinkage ratio (SR)

Shrinkage is expressed by the ratio of the volume of onion slice before and after drying. Shrinkage ratio was calculated at each instant of the drying according to Equation (3) (Dissa, Desmorieux, Savadogo, Segda, & Koulidiati, 2010).

(3) $$\text{SR}\;\left(\%\right)=\frac{\left(V_{i}-V_{f}\right)}{V_{i}}\times 100$$

where, SR (%) is shrinkage ratio, *V*_*i*_ (cm^3^) is the apparent volume of the raw sample before drying, and *V*_*f*_ (cm^3^) is the apparent volume of the sample after drying.

#### 2.5.4 Water hydration capacity (WHC)

Water hydration (absorption) capacity was determined using the centrifugation method described in Beuchat (1977), which is calculated as:

(4) WHC(%)=Initial volume of water−final volume of water

#### 2.5.5 Water activity (*a*_w_)

The water activity of the samples was determined by LabMaster‐*a*_w_ instrument (Novasina AG, CH‐8853 lachen, Switzerland) according to ADOGA (2005).

### 2.6 Determination of chemical properties

#### 2.6.1 Moisture content

The moisture contents of each sample were determined according to the method elaborated in AOAC (2011) 925.09 and calculated as percent loss in weight using Equation (5).

(5) $$\text{Moisture}\;\left(\%\right)=\frac{M_{i}-M_{d}}{M_{i}}\times 100$$

where *M*_*i*_ = intial mass before drying and *M*_*d*_ = final mass after drying.

#### 2.6.2 Total soluble solids (TSS)

Total soluble solids content was determined using refractometer (Model DR201‐95, Germany) as described in Owoso, Aluko, and Banjoko (2000).

#### 2.6.3 pH

The pH value was determined using digital pH meter according to AOAC (2011) official method 981.12.

#### 2.6.4 Titratable acidity (TA)

Titratable acidity was determined using the classical method described in Akhtar, Abbasi, and Hussain (2010). TA was calculated using Equation (6).

(6) $$\text{TA}\;\left(\%\right)=\frac{\text{Titre}\times M\;\text{NaOH}\times F}{0.1\times \text{Weight of sample}}\times 100$$

#### 2.6.5 Vitamin C

Vitamin C content of samples was determined according to the method described in Sadasivam and Manickam (2005).

#### 2.6.6 Pyruvic acid content

Onion pungency is caused by several volatile sulfur‐containing compounds. These compounds are produced when the onion cell is mechanically disrupted, bringing the enzyme alliinase present in the cell cytoplasm into contact with the flavor precursors, *S*‐alk(en) yl‐L‐cysteine sulphoxides. In addition to producing volatile sulphur‐containing compounds, the enzymatic breakdown of the *S*alk (en) yl‐L‐cysteine sulphoxides also produces stoichiometric amounts of ammonia and pyruvic acid. The amount of pyruvic acid generated enzymatically upon homogenization of onion in a blender is thus a good measure of the action of the alliinase on the flavor precursors and has been shown to be correlated with perceived onion pungency (Schwimmer & Weston, 1961). Therefore, pyruvic acid content, as an index of the flavor strength of onions, was measured in mmol/g with a spectrophotometric method (Schwimmer & Weston, 1961).

### 2.7 Sensory evaluation

Sensory analysis of the samples was conducted after rehydrating onion powder (1:2 ratio powder: water v/v) in distilled water for one hour. Each rehydrated sample was coded with three‐digit number and presented to 50 semi‐trained panelists in random order for sensory evaluation of dried onion powder. After tasting each coded sample, the panelists were instructed to rinse their mouth before tasting the next sample. The 5‐point hedonic scale (5 = like extremely, 4 = like moderately, 3 = neither like nor dislike, 2 = dislike moderately, and 1 = dislike extremely) was used to determine sensory acceptability of the samples (Kolapo, Popoola, Sanni, & Afolabi, 2007). The sensory acceptability response variables were color, aroma, taste, and overall acceptability.

### 2.8 Statistical analysis

The main and interaction effects of variety (three levels: Bombay red, Qellafo, and Sweet carolin) and drying temperature (six levels: Fresh, 50, 60, 70, 80, and 90°C) on physical and functional properties (total color change, bulk density, shrinkage ratio, water hydration capacity, and water activity), chemical properties (% moisture, total soluble solids, pH, titratable acidity, vitamin C, and pungency [pyruvic acid]), and sensory characteristics (color, aroma, taste, and overall acceptability) were determined using ANOVA of a 3 × 6 factorial design with three replications. The analysis was completed using the Mixed Procedure of SAS (SAS, 2014), and the validity of model assumptions (normal distribution and constant variance of the error terms) was verified by examining the residuals as described in Montgomery (2017). Independence assumption was assured through randomization of the experiment (Montgomery, 2017). For significant (*p*‐value <0.05) and marginally significant (*p*‐value between 0.05 and 0.1) effects, multiple means comparison was completed using Tukey's Multiple Means Comparison method at the 5% level of significance.

## 3 RESULTS AND DISCUSSION

### 3.1 Effect of temperature and variety on physical and functional properties

The interaction effect of variety and temperature was significant on total color change (ΔE) (Table 1). The change increased with an increase in drying temperature, but the increase was significantly different among onion varieties (Table 2). There was no significant difference among the six temperatures in terms of total color change for Bombay red. However, a small gradual increase with increasing temperature was observed for Qellafo; a more pronounced change was observed for Sweet carolin (Table 2). This implies that, Bombay red was less responsive to heat in terms of total color change than the other varieties. These differences suggest that there is a varietal difference in terms of heat‐sensitive bioactive compounds in addition to browning reactions (Ozgur, Akpinar‐Bayizit, Ozcan, & Yilmaz‐Ersan, 2011).

**Table 1:** ANOVA *p*‐values showing the significance of the main and interaction effects of variety and temperature (Temp) on physical and functional (ΔE = total color change, bulk density, shrinkage ratio, water hydration capacity, water activity) response variables. Significant effects that require multiple means comparison are shown in bold

Source of variation	ΔE	Bulk density	Shrinkage ratio	Water hydration capacity	Water activity
Variety	0.001	**0.001**	**0.025**	**0.001**	0.001
Temp	0.001	**0.001**	**0.001**	**0.001**	0.001
Variety × Temp	**0.049**	0.890	0.597	0.908	**0.001**

**Table 2:** Mean ΔE = total color change, water activity (g/cm^3^), color, aroma, taste, and overall acceptability obtained from the combinations of variety and temperature (Temp in °C)

Variety	Temp	ΔE	Water activity	Color	Aroma	Taste	Overall acceptability
Bombay red	0	NA	0.964^ab^	4.21^bc^	4.00^b^	4.30^ab^	4.30^a^
Bombay red	50	13.9^g^	0.327^c^	3.90^de^	3.61^cde^	3.64^c^	3.90^bc^
Bombay red	60	17.1^g^	0.280^de^	3.69^ef^	3.48^efg^	3.49^cde^	3.82^cde^
Bombay red	70	20.3^fg^	0.262^fgh^	3.49^fgh^	3.45^efg^	3.28^efg^	3.65^f^
Bombay red	80	23.6^efg^	0.250^hi^	3.28^hij^	3.31^fgh^	3.07^hi^	3.54^fgh^
Bombay red	90	27.3^defg^	0.234^jk^	3.00^j^	3.11^hi^	2.80^j^	3.37^ij^
Qellafo	0	NA	0.958^b^	4.48^ab^	4.37^a^	4.44^a^	4.35^a^
Qellafo	50	32.8^cdef^	0.292^d^	4.10^cd^	3.81^bc^	3.55^cd^	3.96^bc^
Qellafo	60	34.8^cde^	0.277^e^	3.88^de^	3.74^bcd^	3.38^defg^	3.84^cd^
Qellafo	70	37.3^bcd^	0.270^ef^	3.65^efg^	3.50^def^	3.33^efg^	3.68^def^
Qellafo	80	41.3^bc^	0.266^efg^	3.36^ghi^	3.42^efg^	3.20^gh^	3.61^f^
Qellafo	90	44.5^ab^	0.251^hi^	3.12^ij^	3.10^hi^	2.91^ij^	3.40^hi^
Sweet carolin	0	NA	0.976^a^	4.60^a^	3.83^bc^	4.42^a^	4.04^b^
Sweet carolin	50	35.3^cde^	0.317^c^	3.64^efg^	3.39^efg^	4.42^a^	3.68^ef^
Sweet carolin	60	39.5^bc^	0.259^fgh^	3.44^fgh^	3.22^gh^	4.17^b^	3.61^f^
Sweet carolin	70	45.2^ab^	0.254^gh^	3.40^fghi^	3.12^hi^	3.41^def^	3.57^fg^
Sweet carolin	80	49.9^a^	0.239^ij^	3.21^hij^	2.94^i^	3.27^fgh^	3.42^ghi^
Sweet carolin	90	51.6^a^	0.222^k^	2.59^k^	2.47^j^	2.81^j^	3.22^j^

*Notes*

NA: Not applicable.

Within each column, means sharing the same letter are not significantly different at the 5% level of significance using Tukey's multiple means comparison method.

Among the other properties, only the main effects, but not the interaction effects, were significant on bulk density, shrinkage ratio, and water hydration capacity (Table 1). The values of all these properties increased with increasing temperature (Table 3), but when varieties are considered, Bombay red and Sweet carolin had similar, but significantly higher bulk density than Qellafo (0.79 g/cm^3^). Results of this study are in line with those reported for tomato (Goula & Adamopoulos, 2005). From commercial perspective, bulk density gives an indication of the relative volume of packaging material required. Drying at high temperature makes a product denser, which leads to requiring stronger packaging material. Furthermore, lower bulk density is desired for powder because it enhances greater ease of dispensability (Udensi & Okoronkwo, 2006) when reconstituted in water.

**Table 3:** Mean bulk density, shrinkage ratio, water hydration capacity, total soluble solids (TSS), and titratable acidity (TA) obtained from the three varieties and six temperatures

Variety	Bulk density	Shrinkage ratio	Water hydration capacity	TSS	TA
Bombay red	0.839^a^	41.9^ab^	1.81^b^	6.59^b^	0.284^b^
Qellafo	0.789^b^	40.3^b^	2.26^a^	6.60^b^	0.318^a^
Sweet carolin	0.823^a^	44.2^a^	1.35^c^	6.93^a^	0.260^c^
Temperature					
0	NA	NA	NA	5.22^d^	0.155^e^
50	0.747^d^	33.7^d^	1.47^c^	6.30^c^	0.286^d^
60	0.789^c^	36.6^cd^	1.61^bc^	6.84^b^	0.299^cd^
70	0.820^bc^	40.8^c^	1.79^b^	7.18^ab^	0.313^bc^
80	0.850^ab^	46.0^b^	2.01^a^	7.48^a^	0.327^ab^
90	0.878^a^	53.5^a^	2.15^a^	7.23^ab^	0.345^a^

*Notes*

NA: Not applicable.

Within each response variable and within each factor, means sharing the same letter are not significantly different at the 5% level of significance using Tukey's multiple means comparison method.

Qellafo variety showed significantly lower shrinkage ratio (40.3) than Sweet carolin, but like that of Bombay red (Table 3). Table 3 indicates that shrinkage ratio gradually increased with increasing drying temperature. The highest ratio (53.5) was obtained at 90°C, and the lowest (33.7) was obtained at 50°C. Results of this study are also in agreement with what was reported in Abasi, Mousavi, Mohebi, and Kiani (2009) where an increase in shrinkage ratio was observed as drying temperature increased from 60 to 90°C. Higher drying temperature resulted in higher moisture removal, and significant tissue structure contraction enhanced tissue modification and overall volume shrinkage; however, the extent of shrinkage differs from variety to variety. Lower shrinkage ratio is more desired by industry for dehydrated products sold by volume.

Water hydration capacity was significantly affected by both variety and temperature (Table 1). Regardless of temperature (no interaction between variety and temperature; Table 1), Qellafo had the highest (2.26%) water hydration capacity followed by Bombay red (1.81%), and Sweet carolin had the lowest (1.35%; Table 3). However, regardless of the variety, water hydration capacity increased with increasing temperature (Table 3). The change in water hydration capacity with an increase in temperature was also reported in Krokida and Maroulis (2000), and in Abasi et al. (2009). As powder absorbs considerably more volume of water, higher water hydration capacity is desirable.

The interaction effect of variety and temperature on water activity was significant (Table 1). As shown in Table 2, water activity values of fresh onion for Bombay red, Qellafo, and Sweet carolin were 0.964, 0.958, and 0.976, respectively. However, water activity values of dried onion powder were less than 0.33 for all three varieties, which is ideal to maintain quality and shelf stability of the powders under proper packaging and ambient storage environment. With an increase in drying temperature, the water activity values decreased in all varieties, but with smaller amount (0.04) for Qellafo (Table 2). Decreasing water activity with increasing temperature was also reported previously (Goula & Adamopoulos, 2005). Storage life of dehydrated agricultural products is highly related with water activity values. Lower value is better because it suppresses microbial growth, biochemical activities, and nonenzymatic browning.

### 3.2 Effects on chemical properties

The interaction effect of variety and temperature was significant on moisture content (Table 4). For fresh onion, the moisture content of Sweet carolin was significantly higher (90.3%) than that of Bombay red (83.3%) and Qellafo (81.7%; Table 5). However, for dried onion, the change in moisture content of the three varieties was mostly similar; all three varieties had less than 8% moisture at 70°C, and at 90°C, the moisture contents were 6.6%, 5.3%, and 4.9% for Sweet carolin, Bombay red, and Qellafo, respectively (Table 5). These results show that with increasing temperature, water loss and solid content increase. The lowest moisture contents in this study are slightly above those reported (2.721% and 1.148% when dried at 60 and 90°C, respectively) in Abasi et al. (2009), which could be due to differences in variety, hold time in the oven, and thickness of slices during drying.

**Table 4:** ANOVA *p*‐values showing the significance of the main and interaction effects of variety and temperature (Temp) on chemical (TSS = total soluble solids, pH, TA = titratable acidity, VC = vitamin C, pungency, and % moisture) response variables. Significant effects that require multiple means comparison are shown in bold

Source of variation	TSS	pH	TA	VC	Pungency	% Moisture
Variety	**0.003**	0.001	**0.001**	0.001	0.001	0.001
Temp	**0.001**	0.001	**0.001**	0.001	0.001	0.001
Variety × Temp	0.633	**0.001**	0.953	**0.001**	**0.001**	**0.001**

**Table 5:** Mean pH, vitamin C, pungency (pyruvic acid content), and % moisture obtained from the combinations of variety and temperature (Temp)

Variety	Temp	pH	Vitamin C	Pungency	% Moisture
Bombay red	0	5.36^a^	4.46^b^	10.07^b^	83.33^b^
Bombay red	50	4.31^c^	1.51^cdef^	5.37^ghij^	7.77^cde^
Bombay red	60	4.27^cd^	1.42^cdefgh^	5.15^hijk^	6.67^cdef^
Bombay red	70	4.20^cde^	1.14^efghi^	4.90^jk^	6.30^def^
Bombay red	80	4.02^fghi^	1.06^ghij^	4.67^jk^	5.93^def^
Bombay red	90	3.69^k^	0.94^ij^	4.45^k^	5.33^ef^
Qellafo	0	5.04^b^	5.03^a^	12.16^a^	81.67^b^
Qellafo	50	4.16^cdef^	1.77^c^	6.93^d^	6.57^cdef^
Qellafo	60	4.11^defg^	1.78^c^	6.73^de^	6.27^def^
Qellafo	70	3.97^ghi^	1.68^cd^	6.40^def^	5.73^def^
Qellafo	80	3.86^ij^	1.52^cde^	6.10^defg^	5.50^ef^
Qellafo	90	3.76^jk^	1.46^cdefg^	5.80^fghi^	4.93^f^
Sweet carolin	0	5.34^a^	4.19^b^	9.17^c^	90.33^a^
Sweet carolin	50	4.32^c^	1.75^c^	5.88^efgh^	9.00^c^
Sweet carolin	60	4.13^defg^	1.34^defghi^	5.22^hijk^	8.23^cd^
Sweet carolin	70	4.05^efgh^	1.11^fghi^	4.99^ijk^	7.80^cde^
Sweet carolin	80	3.92^hij^	1.02^hij^	4.82^jk^	7.27^cdef^
Sweet carolin	90	3.79^jk^	0.68^j^	4.45^k^	6.63^cdef^

*Note*

Within each column, means sharing the same letter are not significantly different at the 5% level of significance using Tukey's multiple means comparison method.

The main effects of variety and drying temperature on total soluble solid (TSS) content were significant, but not the interaction effect (Table 4). The average TSS value of fresh onions was significantly lower than that of dried product (Table 3). The absence of significant changes in TSS value among the last three drying temperatures (70, 80, 90°C) might be because a major portion of the moisture is removed at 70°C. When the varieties are considered (Table 3), Sweet carolin exhibited the highest TSS value (6.93°Brix), but there was no significant difference among the other two varieties. This could be due to enhanced degradation of simple sugars in Bombay red and Qellafo than that in Sweet carolin. Changes in TSS with changes in temperature were also reported in Raj, Subanna, Ahlawat, Gupta, and Huddar (2006), and in Abano, Ma, Qu, and Teye (2011) on drying of onion and garlic at different temperature levels, respectively.

The interaction effect of variety and temperature was significant on pH (Table 4). The pH values of fresh onion varieties were 5.36, 5.04, and 5.34 for Bombay red, Qellafo, and Sweet carolin, respectively (Table 5). Although fresh pH values of the varieties were different, for all varieties, the pH values decreased with an increase in drying temperature, and reached 3.69 to 3.79 at 90°C. However, at 50°C and above, all varieties attained pH < 4.5, which allows controlling the growth of pathogenic food microorganisms except molds and yeasts, but they can be controlled by reduced water activity value. Results in this study are also in agreement with those in Ozgur et al. (2011) who reported a decrease in pH value of leeks after drying. A decrease in pH might be associated with accumulation of more organic acids with the removal of moisture at different drying temperature.

The main effects of variety and temperature on titratable acidity of reconstituted dried onion powder were significant, but not the interaction effect (Table 4). Regardless of variety, as temperature increased, titratable acidity also increased from 0.155 (for fresh) to 0.345 when dried at 90°C (Table 3). Titratable acidity of the three varieties was significantly different from one another, with Qellafo giving the highest and Sweet carolin giving the lowest (Table 3). These results indicate that acidity increases as drying temperature increases, which might be due to the conversion of sugar available in onion into organic acids. Ozgur et al. (2011) and Raj et al. (2006) also reported an increase in titratable acidity of onion and leeks after drying, respectively. The results observed for titratable acidity are in line with the changes in pH as drying temperature increases; however, unlike the changes in titratable acidity, the extent of changes in pH was variety specific. That is, there was significant interaction between variety and temperature for pH, but not for titratable acidity.

Vitamin C is considered as an indicator of food processing quality because of its low stability during thermal treatments (Podsędek, 2007). The interaction effect of variety and drying temperature on vitamin C content was significant (Table 4), and the highest (5.03 mg/100 g) vitamin C was obtained from fresh onion of Qellafo, followed by Bombay red (4.46 mg/100 g) and Sweet carolin (4.19 mg/100 g). Although the magnitude of decrease in vitamin C was not consistent for all varieties, it decreased as drying temperature increased. At 70°C, vitamin C reached 1.68 mg/100 g for Qellafo, 1.14 mg/100 g for Bombay red, and 1.11 mg/100 g for Sweet carolin (Table 5). In other words, the vitamin C content decreased by 66.6%, 74.4%, and 73.5% for Qellafo, Bombay red, and Sweet carolin, respectively, showing Qellafo has relatively better tolerance to drying temperature in terms of conservation of vitamin C content. The negative impact of temperature on vitamin C content of onion powder was also reported by Idah and Obajemihi (2014). Heat degradation of vitamin C is one of the nonoxidative degradation mechanisms that results in the conversion of L‐ascorbic acid to dehydroascorbic acid because the latter, as an intermediate compound, can be easily converted to other compounds having less biological importance (Munyaka, Makule, Oey, Van Loey, & Hendrickx, 2010).

The interaction effect of variety and temperature was significant on pyruvic acid content (Table 4). Pungency (pyruvic acid content) of fresh onion before drying was 12.16, 10.07, and 9.17 μmol/ml for Qellafo, Bombay red, and Sweet carolin, respectively. But at 70°C, the contents were 6.4, 4.9, and 4.99 μmol/ml, which amount to a degradation of 47.4%, 51.3%, and 45.6% for Qellafo, Bombay red, and Sweet carolin, respectively. Adam, Mühlbauer, Esper, Wolf, and Spiess (2000) reported, drying of 2‐mm‐thick onion slices at temperatures of 80°C resulted in 65% loss in pungent pyruvic acid. Our results are also comparable with those of Praveen Kumar, Umesh Hebbar, Sukumar, and Ramesh (2005) who reported a decrease in pungency with an increase in drying temperature. The loss of pyruvic acid content might be attributed to the damage to the cell structure of the onion slices and to subsequent losses of alliinase at elevated temperatures (Sharma & Prasad, 2001).

### 3.3 Effect on sensory acceptability of onion powder

Table 6 shows that the interaction effect of variety and temperature was significant on color, aroma, and taste, and marginally significant on overall acceptability. Color is one of the most important sensory properties of food products. In general, with an increase in drying temperature, the color score values decreased (Table 2), which might be associated with nonenzymatic browning (NEB) that occurred faster at high temperature (Lopez, Mathers, Ezzati, Jamison, & Murray, 2006). Results of this study are also in agreement with the findings of Olalusi (2014) who reported that color of onion slice decreased when drying temperature increased from 50 to 70°C.

**Table 6:** ANOVA *p*‐values showing the significance of the main and interaction effects of variety and temperature (Temp) on sensory response variables. Significant effects that require multiple means comparison are shown in bold

Source of variation	Color	Aroma	Taste	Overall acceptability
Variety	0.001	0.001	0.001	0.001
Temp	0.001	0.001	0.001	0.001
Variety × Temp	**0.001**	**0.001**	**0.001**	**0.080**

Aroma is imparted by volatile compounds and perceived by the odor receptor sites of the smell organ. Particularly, the pungent taste of onion during preparation, processing, and storage is because of the presence of sulfur‐containing volatile oil Allyl‐propyl‐disulphied in the bulb (Mitra, Shrivastava, & Rao, 2012). This compound significantly contributes to the aroma of fresh and dried onion. As indicated in Table 2, with an increase in drying temperature, aroma of onion powder reduced for all varieties, but less in Bombay red and more in Sweet carolin. The same result was observed in different works (Mitra et al., 2011; Olalusi, 2014). This might be associated with the loss of volatile components of powders (Sacilik & Unal, 2005). At a drying temperature of 70°C, Bombay red and Qellafo had better aroma than Sweet carolin (Table 2).

As indicated in Table 2, with an increase in drying temperature, taste of onion powder reduced in all varieties. But, the taste of powder dried at 50°C from Sweet carolin variety was the best. Likewise, at a drying temperature of 70°C, Sweet carolin was rated slightly better than Bombay red and Qellafo (Table 2). However, at 90°C, all onion varieties did not have statistically significant difference. Our results agree with those indicated in Olalusi (2014) where taste of onion slice decreased with increment of drying temperature from 50 to 70°C.

The overall acceptability scores of fresh onions (Table 2) for Bombay red and Qellafo were significantly higher than that of Sweet carolin, and then, it decreased as drying temperature increased for all varieties. Although the overall acceptability of fresh onion of Bombay red and Qellafo varieties was higher, when dried at 90°C, all three varieties have statistically the same overall acceptability.

## 4 CONCLUSIONS

This study showed the presence of variability among onion varieties and the level of drying temperature on physical, chemical, and sensory properties of onion powder. In general, an increase in drying temperature results in reduction of most of the desired properties of onion slices. But a reduction in water activity and pH values of onion powders are desired properties in terms of safety and shelf stability of dried powders. Among the investigated temperatures, drying temperature of 70°C for 5 hr of drying time resulted in comparatively acceptable physical, chemical, and sensory properties. At 70°C, Qellafo resulted in superior results for most of the parameters as compared to the other two varieties. Therefore, Qellafo is recommended as a suitable onion variety when production of dehydrated onion product is desired.

## CONFLICT OF INTEREST

The authors declare that there is no conflict of interest.
